# Supplementary material for: Stacking Model‐Based Classifiers for Dealing With Multiple Sets of Noisy Labels
Source: Biom J. 2025 Mar 12;67(2):e70042. doi: 10.1002/bimj.70042 (PMC11898607; doi:10.1002/bimj.70042)
Supplement: Supplementary file 1 — Supporting Information [file BIMJ-67-e70042-s001.zip › density-based-ensemble-model-main/competitors_real_data/netlab-master/netlab-master/help/plotmat.htm]

Netlab Reference Manual plotmat


# plotmat

## Purpose

Display a matrix.

## Synopsis

```
plotmat(matrix, textcolour, gridcolour, fontsize)
```

## Description

`plotmat(matrix, textcolour, gridcolour, fontsize)` displays the matrix
`matrix` on the current figure. The `textcolour` and `gridcolour`
arguments control the colours of the numbers and grid labels respectively and
should follow the usual Matlab specification.
The parameter `fontsize` should be an integer.

## See Also

`conffig`, `demmlp2`

---

**Pages:**
Index


---

Copyright (c) Ian T Nabney (1996-9)
